# Measuring the effects of socioeconomic factors on mental health among migrants in urban China: a multiple indicators multiple causes model

**DOI:** 10.1186/s13033-016-0118-y

**Published:** 2017-01-06

**Authors:** Ming Guan

**Affiliations:** Family Issues Center at Xuchang University, School of Business, Xuchang University, Road Bayi 88, Xuchang, Henan China

**Keywords:** Socioeconomic factors, Mental health, GHQ-12, Rural–urban migrants, Urban China, MIMIC

## Abstract

**Objectives:**

Since 1978, rural–urban migrants mainly contribute Chinese urbanization. The purpose of this paper is to examine the effects of socioeconomic factors on mental health of them. Their mental health was measured by 12-item general health questionnaire (GHQ-12).

**Methods:**

The study sample comprised 5925 migrants obtained from the 2009 rural-to-urban migrants survey (RUMiC). The relationships among the instruments were assessed by the correlation analysis. The one-factor (overall items), two-factor (positive vs. negative items), and model conducted by principal component analysis were tested in the confirmatory factor analysis (CFA). On the basis of three CFA models, the three multiple indicators multiple causes (MIMIC) models with age, gender, marriage, ethnicity, and employment were constructed to investigate the concurrent associations between socioeconomic factors and GHQ-12.

**Results:**

Of the sample, only 1.94% were of ethnic origin and mean age was 31.63 (SD = ±10.43) years. The one-factor, two-factor, and three-factor structure (i.e. semi-positive/negative/independent usefulness) had good model fits in the CFA analysis and gave order (i.e. 2 factor>3 factor>1 factor), which suggests that the three models can be used to assess psychological symptoms of migrants in urban China. All MIMIC models had acceptable fit and gave order (i.e. one-dimensional model>two-dimensional model>three-dimensional model).

**Conclusions:**

There were weak associations of socioeconomic factors with mental health among migrants in urban China. Policy discussion suggested that improvement of socioeconomic status of rural–urban migrants and mental health systems in urban China should be highlighted and strengthened.

**Electronic supplementary material:**

The online version of this article (doi:10.1186/s13033-016-0118-y) contains supplementary material, which is available to authorized users.

## Background

During the past three decades, an estimated 200 million rural residents have migrated into urban China. These migrants in urban China may experience higher level of psychological disorders due to the obscure role of trade unions [1], discrimination experience [2], and socioeconomic inequality in opportunities and the lack of social support. Rural-to-urban migrant workers in China had experienced various forms of employment stigmatization including labelling, stereotyping, separation, status loss and discrimination [3]. Migrant adolescents experienced perceived discrimination in China [4]. Compared with the Chinese general population, they had low use rate of mental health services [5, 6].

These migrants in urban China experience higher level of psychological disorders than the general population. Rural–urban migrant workers manifested a high prevalence of both life stress and work stress [7] and experienced acculturative stress [8]. The mental health status of young migrant workers was poorer than that of their local counterparts [9]. It indicated a higher prevalence of depression symptoms among migrant workers comparing to general population [10]. Chinese migrant workers had more severe psychological symptoms than the general population, and thus, appear to experienced higher level of psychological distress [11]. Seen as a major public health issue, loneliness was prevalent in Chinese service industry rural-to-urban migrant workers [12].

Rural-to-urban migrants had their own mental health trajectories [13]. The socioeconomic factors, including age and marital status, were influential factors for depression scores among migrant workers [14]. A study conducted in Guangzhou city, China indicated that different vocation, sex, and working years might interfere with the psychological states among migrant workers [15]. With regards to employment, mental health status of unemployed migrant workers in Eastern China was poorer than the national adult norm [16]. Studies conducted in Shanghai, China suggested that financial and employment difficulties [17] and marital status [18] contributed substantially to the mental health of migrant workers. Regarding age, there was mental health gap between older and younger migrants [19]. The self-reported mental health status in migrant workers was poor and was associated with age [20]. Migrant children reported more internalizing and externalizing mental health problems and lower life satisfaction than local peers [21]. Considering gender, rural-to-urban female migrant workers had a lower quality of life compared to the general population [22]. Another study conducted in Shenzhen, China indicated that 24% of female migrant workers had poor mental health [23].

Also, the early studies reported socioeconomic factors contributed to mental health status in the international academic communities. For example, the importance of psycho-social factors was emphasized in determining common mental disorders in the general practice [24]. Ethnic disparities in health started early in life [25]. Work could play in enhancing mental well-being [26]. Permanent employees in future temporary employment had poorer mental health than stable employees [27]. Socioeconomic disparities existed for older adults, and poor oral health diminished quality of life [28]. Hence, it may be an interesting matter to consider the link between socioeconomic factors and mental health in migrants in urban China.

Mental health of migrant workers is an important public health issue in China. Compared to overseas studies, children of migrant workers suffered from symptoms of separation anxiety, depression and generalized anxiety disorder [29]. The migration was an important risk factor for child depression [30]. Labor out-migration had important consequences for the mental health in migrant-sending communities [31]. Poor mental health increased likelihood of smoking [32], current smoking behaviors [33], and suicides [34] among rural-to-urban migrants.

The general health questionnaire-12 (GHQ-12) could be used to detect some psychiatric illnesses among rural–urban migrants. This study aims to provide a better insight into the associations of socioeconomic factors with mental health among migrants in urban China using the GHQ-12. The multiple indicators multiple causes (MIMIC) model was used to integrated socioeconomic variables. A large sample from a publicly available survey dataset is adopted here.

## Methods

### Data source

2009 rural-to-urban migrants survey (new household and old household) from 2009 rural–urban migration in China project (RUMiC) was used here which were supported by the Institute for the Study of Labor. The RUMiC database is being constructed by a team of researchers from Australia and China. See the RUMiC Project’s homepage (http://rumici.anu.edu.au/joomla/), the rural–urban migrants were surveyed in the 15 cities, including eight cities in coastal regions, five cities in central inland regions and two cities in the west. The content validity of the measure was assessed by the RUMiC expert panel. All items were agreed upon so that they were relevant to the context of migrants in urban China.

### Subjects

Among total new and old households, 5925 subjects replied to GHQ-12 contained in 2009 RUMiC questionnaire. With regards to marital status, 58.80% were married, 0.76% were remarried, 0.64% were cohabited, 1.59% were divorced, 0.76% were widowed, and 37.45% were never married. Regarding ethnicity (n = 5889), 98.66% were Han ethnicity, 0.32% were Zhuang ethnicity, 0.61% were Hui ethnicity, 0.08% were Uyghur ethnicity, 0.05% were Yi ethnicity, 0.19% were Miao ethnicity, and 0.08% were Manchu ethnicity. Considering current work status (n = 5916), 95.81% were employed, 0.14% were reemployed retiree, 1.10% were unemployed, 0.27% were retired, 0.83% were housemakers, 1.45% were family business helper without pay, 0.03% lost work capabilities, 0.20% were in school student/preschool child, and 0.17% were awaiting job assignment/further education/dropout student. Their height, weight, and BMI were 165.98 (SD = ±7.21) cm, 60.77 (SD = ±9.93) kg, and 22.01 (SD = ±2.97) kg/m^2^, respectively. The average number of years of formal education was 9.41 (SD = ±2.60).

### Measures

GHQ-12 was contained in 2009 RUMiC questionnaire as a measure of mental disorder. GHQ-12 was a useful screening instrument for the detection of depression [35], psychological well-being [36], and mental disorders [37, 38]. GHQ-12 worked as an effective tool to judge the mental health in the western countries [39] and Asian population [40]. Prior studies reported one-factor model [41], two-factor model [42], eight-item two-factor model [43], and three-factor model [44] were identified. With high internal consistency [45], GHQ-12 measured both positive [46] and negative aspects of mental health with some satisfactory psychometric properties [47, 48]. Also, factorial structures of the Chinese version of the GHQ-12 had been identified in a specific population [49].

### Statistical analysis

There are six stages to the analysis. The first stage used the descriptive statistics to analyze socioeconomic factors. In the second stage, tetrachoric correlations were estimated between the items among GHQ-12. In the third stage, logistic regression was used to analyze how socioeconomic factors influence mental symptoms. Among socioeconomic variables, age was a continuous variable. The other variables were dichotomized into gender (male = 1, female = 0), marriage (married = 1, others = 0), ethnicity (Han = 1, others = 0), and employment (employed = 1, others = 0). In the fourth stage, principal component analysis (PCA) with varimax rotation was employed to examine the factor structure. The unidimensional model, two-dimensional model (positive and negative questions), and the model conducted by PCA consisted in the exploratory factor analysis (EFA). The subsequent confirmatory factor analysis (CFA) was computed on the basis of EFA. In order to adopt PCA and logistic regression, each item on the GHQ-12 was dichotomized into the two values: 0 = “absence of the symptom” and 1 = “presence of the symptom”. In the fifth stage, three confirmatory factor models to test the global fit of the factor structure of GHQ-12 were estimated. The final stage was to compute the MIMIC models based on the CFA.

CFA was performed in order to find the most fitted model. Here, three MIMIC models should be considered where socioeconomic variables were incorporated in the CFA models. Here, the main index was used to measure goodness of fit: root mean square error of approximation (RMSEA). RMSEA should have values below 0.10 for acceptable fit, and below 0.05 for good fit [50]. Analyses were completed using Stata 14.0 (Stata Corporation, Texas, USA).

## Results

### Sample characteristics

The subjects of this study were 5925 migrants. The sample contained 3557 males and 2368 females with an average age of 31.63 (SD = ±10.43) ranging from 16 to 78 years. As shown in Table 1, a high proportion (95.81%) of the sample was employed. Additionally, 98.06% of was Han ethnicity, and 1.94% belongs to ethnic minority.

**Table 1:** Socioeconomic characteristics of rural–urban migrants in China (n = 5925)

	Total	Male	Female	Ch2	p value
Age	31.63 ± 10.43	31.15 ± 9.92	31.95 ± 10.75	111.9323	0.000
Marital status (%)				5.5138	0.019
Married	58.80	34.57	24.24		
Other	41.20	25.47	15.73		
Current work status (%)				61.6141	0.000
Employed	95.66	58.45	37.22		
Other	4.34	1.59	2.75		
Ethnicity (%)				5.2878	0.021
Han ethnicity	98.06	58.67	39.39		
Ethnic minority	1.94	1.37	0.57		

* p values were derived from Chi^2^ test

Table 1 shows that significant gender differences among the rural–urban migrants were found in the age, marital status, current work status, and ethnicity (p < 0.05). The mental health of the migrants could be speculated to be poor (Additional file 1).

### Correlations

See Table 2. Tetrachoric correlations coefficients were more than 0.30. Among them, the correlations ranged from 0.319 to 0.773, and no coefficients had a negative value. The correlation coefficient between GHQ 10 and 11 was maximum. Most correlation coefficients of significance was used as a standard for assuming good scale-internal consistency.

**Table 2:** Tetrachoric correlations between the GHQ-12 (n = 5925)

	1	2	3	4	5	6	7	8	9	10	11
GHQ 1											
GHQ 2	0.545										
GHQ 3	0.478	0.386									
GHQ 4	0.422	0.346	0.563								
GHQ 5	0.458	0.637	0.364	0.311							
GHQ 6	0.435	0.536	0.414	0.376	0.683						
GHQ 7	0.431	0.474	0.453	0.451	0.516	0.470					
GHQ 8	0.400	0.324	0.457	0.470	0.319	0.348	0.431				
GHQ 9	0.461	0.583	0.399	0.380	0.610	0.661	0.526	0.466			
GHQ 10	0.378	0.466	0.437	0.480	0.540	0.666	0.495	0.430	0.687		
GHQ 11	0.410	0.464	0.437	0.437	0.504	0.597	0.555	0.458	0.603	0.773	
GHQ 12	0.383	0.464	0.361	0.408	0.520	0.455	0.654	0.428	0.523	0.510	0.575

Values in the cells are significant, p < 0.001

### Logistic regression

See Table 3. Age had significantly positive relationships with GHQ item 2, 3, 5, 6, 7, 10, 11, and 12. Gender had significantly negative relationships with GHQ item 1, 2, 3, 4, 8, 10, and 11. Employed status had significantly negative relationships with GHQ item 1, 3, and 4. Married status had significantly negative relationships with GHQ item 3, 7, 8, and 12. Han majority had significantly negative relationships with GHQ item 5.

**Table 3:** Odds ratios of logistic regression of GHQ items on socioeconomic factors

	GHQ1	GHQ2	GHQ3	GHQ4	GHQ5	GHQ6	GHQ7	GHQ8	GHQ9	GHQ10	GHQ11	GHQ12
Age	0.01	0.02**	0.02***	0.00	0.02***	0.02**	0.02***	-0.01	0.01	0.03***	0.04***	0.03***
Gender (male = 1)	−0.26**	−0.37***	−0.34***	−0.53***	−0.03	−0.08	−0.12	−0.46***	−0.16	−0.29*	−0.30*	−0.00
Employment												
Others (=ref.)												
Employed	−0.47**	−0.42	−0.61***	−0.47*	−0.33	−0.21	0.04	−0.31	−0.44	−0.39	−0.31	0.19
Marriage												
Others (= ref.)												
Married	−0.08	0.05	−0.38***	0.02	0.01	0.18	−0.32***	−0.41***	−0.21	−0.23	−0.33	−0.60***
Ethnicity												
Others (= ref.)												
Han majority	−0.23	−0.23	−0.30	0.48	−0.61*	0.07	−0.11	−0.30	0.19	1.41	0.50	−0.43
_cons	−1.32***	−2.51***	−1.78***	−2.15***	−1.54***	−3.50***	−2.22***	−0.63	−2.81***	−4.92***	−4.43***	−2.34***

* p < 0.1

** p < 0.05

*** p < 0.01

### Factor structure

There were three factor structure models for GHQ-12. They were one-factor model, two-factor model structured in positive and negative dimensions, and the model computed by PCA. In the one-factor model, alpha was 0.7569. In the two-factor model, scale reliability alpha coefficients were 0.6344 for positive items (GHQ 1, 3, 4, 7, 8, and 12) and 0.6815 for negative items (GHQ 2, 5, 6, 9, 10, and 11). The internal consistency of the GHQ-12 assessed by Cronbach’s alpha was 0.7569.

The PCA and varimax rotation method were used to create the third factor structure model. Kaiser-Meyer-Olkin measure of sampling adequacy was 0.8552, which indicates adequate sample size for the factor analysis. Bartlett’s test of sphericity was significant (*χ* ^2^ = 18593.875, df = 66, p < 0.001). PCA with varimax rotation solution showed three components. This study was in line with Kaisers rule [51] which using eigenvalues >1 determine the number of factor in the solution. Eigen values were 3.409, 1.173 and 1.044.

See Table 3. A three-dimensional structure was identified in the EFA. Factor 1, labeled as semi-positive health, which included item 1, 5, 6, 7, 8, 9, and 12 of the GHQ-12. Factor 2, labeled as negative health, which contained item 3 and 4. Factor 3, labeled as Independent usefulness, which contained item 2, 10, and 11. The three factors together explained 46.88% of the variance (Table 4).

**Table 4:** Factor structure with principal component analysis

	Factor 1	Factor 2	Factor 3
GHQ1	0.4898		
GHQ2	0.5156		−0.4906
GHQ3	0.4657	0.4750	
GHQ4	0.4491	0.4884	
GHQ5	0.5882		
GHQ6	0.5622		
GHQ7	0.5782		
GHQ8	0.4524		
GHQ9	0.5973		
GHQ10	0.5705		0.4592
GHQ11	0.5405		0.4680
GHQ12	0.5562		

Extraction method: principal component analysis

Rotation method: varimax with Kaiser normalization

Data shown as Eigenvalues (coefficient)

Blanks represent abs (loading) <0.4

### Confirmatory factor analysis

The one-factor model, two-factor model (positive vs. negative items),and three-factor model were analyzed. They provided acceptable fit for the data (RMSEA = 0.069, 0.058, and 0.065), respectively (Additional files 2, 3 and 4). All fit indexes of the models had acceptable goodness of fits, while the two-factor model competed with the other two models.

### MIMIC model

Based on the logistic regressions, three multiple indicators multiple causes (MIMIC) models integrated three CFA models were applied to test the associations between socioeconomic factors and mental health. Hence, one-dimensional MIMIC model, two-dimensional MIMIC model, and three- dimensional MIMIC model were constructed.

**Fig. 1:**
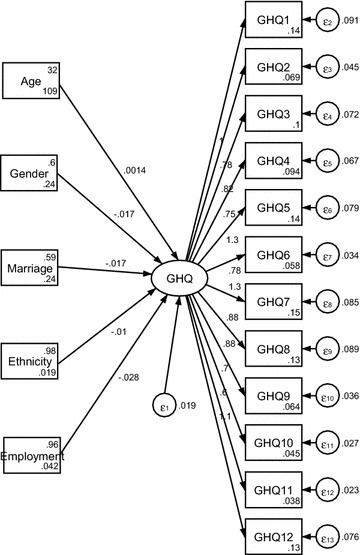
One-dimensional MIMIC model

**Fig. 2:**
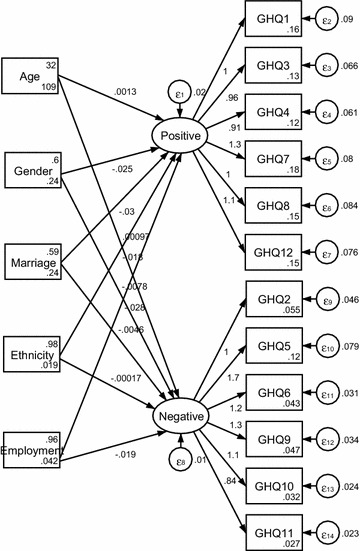
Two-dimensional MIMIC model

**Fig. 3:**
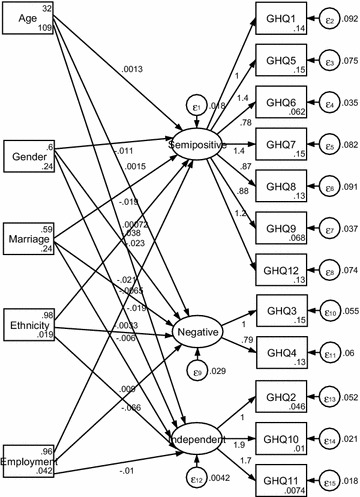
Three-dimensional MIMIC model

See Figs. 1, 2 and 3. The one- dimensional MIMIC model provided an acceptable fit for the data (RMSEA = 0.051). The two-dimensional MIMIC model provided an acceptable fit for the data (RMSEA = 0.069). The three-dimensional MIMIC model provided an acceptable fit for the data (RMSEA = 0.085). Hence, the model with the best fit in the MIMIC was the one- dimensional model, followed by the two-dimensional MIMIC model and three-dimensional model derived from the CFA.

See Fig. 1. Among socioeconomic factors, only age positively influenced GHQ, while gender, marriage, ethnicity, and employment negatively affected migrants’ mental health in a small reach.

See Fig. 2. Among socioeconomic factors, only age and ethnicity positively influenced positive mental health, while gender, marriage, and employment negatively affected it. All the five socioeconomic factors negatively affected negative mental health.

See Fig. 3. Among socioeconomic factors, gender, marriage, and employment negatively influenced semi-positive health, negative health, and independent usefulness, while age positively influenced them. Ethnicity positively influenced Independent usefulness, while it negatively affected Semi-positive health and negative health.

## Discussion

The findings from present study showed that the GHQ-12 of migrants in urban China was a valid measure of psychological disorder. Tetrachoric correlation coefficients between the GHQ dimensions were less than 0.8. In the logistic regression, the main socioeconomic factors had significantly relationships with GHQ items. All fit indexes of the three CFA and MIMIC models had acceptable goodness of fit. But, the one-dimensional model had best fit in the MIMIC models. The small coefficient values of socioeconomic factors showed quantitatively of little importance, while they were full of special clinical interest and practical use. Clinically, the CFA and MIMIC models could play an important role in monitoring the mental health care and utilization.

This study specified how socioeconomic factors were linked to psychological well-being with dimensional models. The association between socioeconomic factors and mental health in China may differ from other countries in a migrant setting. Compared with Padrón’s et al. [52] three-dimensional model derived from the EFA, this three-dimensional model had better fit after computation. But, the coefficients between socioeconomic factors and latent variables were small which suggested the associations had a bit significant impact on an migrants’ non-psychotic psychiatric problems. These findings induced us to guess there might be discriminations on rural–urban migrants which damaged mental health of them. In fact, most of migrants come from poverty-stricken remote and border areas. Language and cultural barriers made them difficult to access to health service. Additionally, the results here confirmed that the GHQ-12 could best be thought of as a multidimensional scale that assesses several distinct aspects of mental disorder, rather than just a unitary screening measure [53]. The empirical study suggested assessment of dimensional structure of the specific population group could help target the clinical angles. Especially, risk factors of poor mental health from job loss, unstable marriage, ageing process, and ethnic minority status have received less nursing attention in regard to physical health effects.

This study accords with the early results linking gender with mental health. For instance, there was gender difference in the emotional advantages of marriage conflicting with mental illness [54]. Mental health was diagnosed on general health questionnaire and found that work long time and gender were associated with mental disorders [55].

This study is consistent with the early results linking marital status with psychiatric impairment. For example, divorced marital status was related to psychiatric impairment [56]. The influence of marriage on mental health benefits varied across the life course [57]. In modern China, equality was still lacking in common households facing negative health stressors. But, marriage equality was related to improved health outcomes [58]. This can be explained by the result that the mental health among older married couples was correlated using the GHQ-12 to measure mental health [59].

This study is also in line with the early results linking employment with mental health state. For instance, transition from unemployment into permanent employment could contribute to improved health [60]. But, change from permanent to temporary employment increased the probability of developing mental health problems [61]. Movements among paid employment, non-employment, and unemployment had an impact on mental health [62]. Unemployment might concur with poor mental health [63, 64]. Precarious employment was a risk factor for subsequent development of mental health problems [65]. There was an increase in poor mental health among immigrant workers experienced deterioration in employment conditions [66]. This can be explained by the result that there might be physical and mental health benefits associated with obtaining or keeping employment [67]. Multiple spells of unemployment and leaving employment for reasons were associated with reduction in mental health [68].

The current study replicated the effort linking ethnicity to health. The current study could be seen as a natural continuation of the work done by Kerson [69], Nazroo [70], and Bracken et al. [71]. This finding was consistent with the results reported by Kim et al. [72] who reported ethnic minority immigrants in Canada might be more vulnerable to social changes and post migration settlement. In addition, ethnicity was an important predictor of mental health care use in young children [73]. With regards to the effect of ethnicity on mental health, the current results were not in line with the findings from other studies which ethnicity could not efficiently predict adult mental health [74] and accounted for differences in the health-related quality of life of international migrants [75]. The findings were consistent with the view that ethnicity itself was not related to disparities in mental health [76]. In China, rural–urban migrants live in social disadvantage. Social disadvantage is more common in rural-to-urban migrants than in urban locals due to household registration discrimination. This suggested that the higher prevalence of mental disorders among rural-to-urban migrants cab be explained partly by their disadvantaged social position. Against Han majority, ethnicity was likely to strengthen mental status in rural-to-urban migrants.

From the perspective of clinical practice, this study highlighted the role of socioeconomic factors in the mental health. In line with the previous results, socioeconomic factors could act as lens to monitor mental health. Improving employment opportunities could promote mental health [77]. Employment was suggested to cure people experiencing a serious mental health illness [78]. Perceived discrimination was associated with worse mental health [79]. Ethnicity might be one of psychosocial barriers to care [80] experienced by gender [81]. Confirmatively, the socioeconomic status did not influence access to services in the general medical sector in the older adult population [82]. Hence, mental health care partial to rural–urban migrants sponsored by local and central governments should be promoted.

Mental health systems in urban China are facing migrant challenges. This study promotes flaws discovery at the system level, which shows improvement in migrant livelihood surpasses the supply of mental health care. Reorganizing mental health systems to render better, affordable, and available mental health care requires transformation in the process of generating and applying among the knowledge mental health systems. Simultaneously, China’s government should better mental health infrastructure and promote innovation on the basis of mental change. In order to protect migrants with mental illness, incremental investment of mental health should be valued as an important tool to develop, strengthen, advance and shape the mental health systems. Legislation and policy of the mental health also need attention to upgrade the mental health service for rural–urban migrants.

## Limitations

The main limitation lies in cross-sectional data. The current study used cross-sectional data rather than panel data can not report the dynamic laws of change of mental health and socioeconomic status among migrants in urban China. Rapid economic growth and frequent release of reforming policies might conflict with the findings in this study. Another limitation may be misunderstanding of the questionnaire due to low-level education of the migrants. Often, it is the first time for the subjects to be interviewed. Without practical interpretation, expectations and emotions in heart possibly were substituted for the objective answers. Other socioeconomic factors, such as religious practice, income, and education attainment, have not been included here. These limit the result generalization to more larger population.

## Future direction

Future researchers should consider time and governmental reflections to the mental health of rural–urban migrants. Also, these findings could be further tested in a rural and urban population to determine the possible effect of socioeconomic factors on mental health in the future.

Moreover, some results confounded by other socioeconomic factors need be explored in the sample. For example, whether retirement was associated with psychological distress or not depends on gender [83]. The relationship between ethnicity and mental health might be confounded by other socioeconomic or health differences [84]. Understanding the effects of unemployment on mental health required consideration of the interactions among gender, family responsibilities, and social class [85]. The joint impact of age and gender regulated the contribution of psychological resources to health [86] and interacted with emotional health [87].

## Conclusion

The present study has the virtue of pioneering research into the evaluation of mental health among rural–urban migrants using GHQ-12. This study reported three MIMIC models reflecting the association between socioeconomic factors and mental health based on the three CFA models. The good-for-fit of structural equation models reported that the models could be accepted. The results may be generalized in China due to data reflecting Chinese urban settings. The knowledge generated here may promote clinical and practical innovation of mental health. This study also confirmed common sense of factorial structure helped to clinical practice because one and two-factor model emerged from basic understanding of GHQ-12 have the acceptable fits in the sample. This study also confirmed that the incidence, prevalence and presentation of mental disorders differ by age, gender, marriage, ethnicity, and employment. Without discussing interactions among socioeconomic factors, this study has its limitations.

Regarding policy design and implementation, it is necessary to assess dimensional structure of MIMIC GHQ-12 for clinical practice, especially for mental health care and service. As a result, the findings have practical implications that the knowledge generation and application of mental health among the migrants will benefit renovation and change of mental health systems in China.

## Additional files

**Additional file 1.** Computation of mental health of migrants.

**Additional file 2.** One-factor CFA model.

**Additional file 3.** Two-factor CFA model.

**Additional file 4.** Three-factor CFA model.

## Abbreviations

GHQ-12: 12-item general health questionnaire
RUMiC: the rural-to-urban migrants survey
PCA: principal component analysis
EFA: exploratory factor analysis
CFA: confirmatory factor analysis
MIMIC: multiple indicators multiple causes
