# Supplementary material for: Measuring the effects of socioeconomic factors on mental health among migrants in urban China: a multiple indicators multiple causes model
Source: Int J Ment Health Syst. 2017 Jan 6;11:10. doi: 10.1186/s13033-016-0118-y (PMC5217273; doi:10.1186/s13033-016-0118-y)
Supplement: Supplementary file 1 — Additional file 1. Computation of mental health of migrants. [file 13033_2016_118_MOESM1_ESM.doc]

Computation of mental health of migrants

Table. Statistical characteristics in GHQ-12 items.

| Item no. | Obs. | Mean | Std. Dev. | Min | Max |
| --- | --- | --- | --- | --- | --- |
| GHQ1 | 5925 | .126 | .332 | 0 | 1 |
| GHQ2 | 5925 | .060 | .238 | 0 | 1 |
| GHQ3 | 5925 | .094 | .291 | 0 | 1 |
| GHQ4 | 5925 | .085 | .279 | 0 | 1 |
| GHQ5 | 5925 | .125 | .331 | 0 | 1 |
| GHQ6 | 5925 | .049 | .215 | 0 | 1 |
| GHQ7 | 5925 | .132 | .339 | 0 | 1 |
| GHQ8 | 5925 | .118 | .323 | 0 | 1 |
| GHQ9 | 5925 | .054 | .225 | 0 | 1 |
| GHQ10 | 5925 | .037 | .190 | 0 | 1 |
| GHQ11 | 5925 | .031 | .174 | 0 | 1 |
| GHQ12 | 5925 | .114 | .318 | 0 | 1 |

∵ Mean GHQ1+ Mean GHQ2+…+ Mean GHQ12=1.025>1,

∴The mental health of the migrants could be speculated to be poor.
