# Supplementary material for: Measuring the effects of socioeconomic factors on mental health among migrants in urban China: a multiple indicators multiple causes model
Source: Int J Ment Health Syst. 2017 Jan 6;11:10. doi: 10.1186/s13033-016-0118-y (PMC5217273; doi:10.1186/s13033-016-0118-y)
Supplement: Supplementary file 2 — Additional file 2. One-factor CFA model. [file 13033_2016_118_MOESM2_ESM.doc]

Fig. 1

One -factor CFA model
